# Resting and exercise arterial dysfunction in anthracycline-treated adult survivors of childhood cancers

**DOI:** 10.1186/s40959-018-0035-0

**Published:** 2018-12-22

**Authors:** Vivian Wing-yi Li, Anthony Pak-yin Liu, Karin Kar-Huen Ho, Jeffrey Ping-Wa Yau, Daniel Ka-leung Cheuk, Yiu-fai Cheung

**Affiliations:** 1 Division of Paediatric Cardiology, Department of Paediatrics and Adolescent Medicine, Queen Mary Hospital, The University of Hong Kong, 102, Pokfulam Road, Hong Kong, China; 2 Department of Paediatrics and Adolescent Medicine, Princess Margaret Hospital, Hong Kong, China; 3 Department of Paediatrics, Queen Elizabeth Hospital, Hong Kong, China

## Abstract

**Background:**

Emerging evidence suggests potential arterial damage with the use of anthracycline-based chemotherapeutic regimens. We determined arterial function at rest and during exercise in anthracycline-treated adult survivors of childhood cancers.

**Methods:**

Ninety-six adult survivors (54 males) aged 25.0 ± 5.9 years and 60 (30 males) healthy controls were studied. Central systolic blood pressure (cSBP) and radial augmentation index (rAI) was determined by applanation tonometry. Carotid arterial stiffness and intima-media thickness (IMT) were assessed using high-resolution ultrasound.

**Results:**

At rest, survivors had significantly greater carotid IMT (*p* < 0.001) and stiffness index (p < 0.001), and higher cSBP (*p* = 0.037), rAI (*p* = 0.004) and rAI adjusted for a heart rate of 75/min (*p* = 0.009) than controls. At submaximal supine exercise testing, survivors had significantly greater percentage increase in carotid stiffness than controls (*p* < 0.001). Among survivors, 32 and 53% had respectively carotid IMT and exercise stiffness index exceeding normal (> + 2SD of controls). The slopes of increase in carotid IMT (*p* < 0.001) and exercise-induced changes in carotid stiffness (*p* < 0.001) with age were significantly greater in survivors than controls. Multivariate analysis revealed carotid IMT (β = 0.32, *p* < 0.001) to be an significant correlate of dynamic percentage increase in stiffness index during exercise.

**Conclusions:**

Arterial dysfunction is evident at rest and worsens during exercise in anthracycline-treated adult survivors of childhood cancers.

## Background

Left ventricular dysfunction is well documented in anthracycline-treated long-term survivors of childhood cancers [1–3]. Optimal performance of the left ventricle depends not only on its intrinsic properties but also on normal functioning of the systemic arterial system, which constitutes the LV afterload and influences ventriculo-arterial interaction [4, 5]. Recent large epidemiologic studies reaffirmed the increased risk of cardiovascular [6–8] and cerebrovascular [9] events in long-term survivors of childhood cancers. Importantly, these findings implicate the possibility of an early onset of arterial dysfunction, which may predispose to development of major vascular events, in these long-term survivors.

Emerging evidence suggests potential arterial damage with the use of anthracycline-based chemotherapeutic regimens. Kaushal et al. has demonstrated in-vitro toxicity of anthracyclines on endothelial cells [10]. Adults undergoing treatment for breast cancer, lymphoma or leukaemia were found to have increase in aortic stiffness early after anthracycline treatment [11]. There is, however, a paucity of data on long-term alterations of arterial structure and function in adult survivors of childhood cancers. Furthermore, studies on arterial function in cancer survivors reported to date were limited by the assessment of only the resting arterial function.

The potential clinical relevance of arterial mechanics during exercise is increasingly recognized. In patients with coronary artery disease, isometric handgrip exercise has helped to unmask the increase in arterial stiffness [12]. In patients after arterial switch operation for complete transposition of the great arteries, we have found associations between reduced aortic distensibility and strain at submaximal exercise and dilation of the neoaortic sinus [13]. In cancer survivors, dynamic changes of arterial stiffness during exercise have not been studied.

In the present study, we aimed to assess the resting and exercise arterial function in anthracycline-treated adult survivors of childhood cancers and to determine factors associated with alterations in arterial structure and function in this at-risk population.

## Methods

### Subjects

Ninety-six anthracycline-treated adult survivors of childhood cancers who had been off treatment for at least 5 years were recruited from three public hospitals in Hong Kong. Exclusion criteria included history of congenital heart disease and presence of syndromal disorders and documented cardiomyopathy. Systemic ventricular dysfunction is associated with sympathoadrenal activation and activation of the renin-angiotensin system [14, 15]. In patients with clinical heart failure, increased arterial stiffness is well documented [16, 17]. To avoid the confounding influence of heart failure on arterial functional assessment, we have therefore excluded survivors with cardiomyopathy. The following data were retrieved from the case notes: diagnosis, age at and year of diagnosis, date of start and completion of chemotherapy, cumulative doses of anthracycline, and the need for cardiac irradiation. Sixty age-matched healthy subjects including volunteers and healthy siblings of survivors with no clinical history of cardiovascular diseases were recruited as controls.

The body weight and height were measured, and body mass index and body surface area were calculated accordingly. All subjects underwent assessment of vascular function at rest and during supine bicycle exercise testing as described below. All subjects gave written informed consent to participate in this study, which was approved by the Institutional Review Board of the University of Hong Kong/ Hospital Authority West Cluster, Hong Kong. All of the methods were performed in accordance with the regulations and the relevant guidelines.

### Assessment of vascular structure and function

#### Ultrasound assessment of carotid arteries

High-resolution ultrasound imaging of the right carotid artery was performed using the linear M12 L (5.6–14.0 MHz) probe interfaced with the Vivid 7 ultrasound system (GE Medical System, Horten, Norway). The average value of indices from three cardiac cycles was obtained for statistical analysis.

The intima-media thickness (IMT) of the far wall of right common carotid artery, at about 1 cm proximal to its bifurcation, was measured at end-diastole. The resting and exercise stiffness indices of the right common carotid artery were determined by relating the changes in blood pressure to changes in arterial dimension during the cardiac cycle, calculated as ln (SBP- DBP) / [(Ds-Dd)/Dd] [18], where Dd and Ds are end-diastolic and end-systolic (Ds) carotid arterial dimensions, respectively. The systolic (SBP) and diastolic (DBP) blood pressures of the right arm were measured by an automated oscillometric device (Dinamap, Critikon, Tampa, FL, USA).

#### Radial arterial waveform

To estimate the central aortic pressure, the right radial arterial pulse waveform was obtained by applanation tonometry (HEM 9000-AI, Omron-Healthcare, Kyoto, Japan) [19]. Based on the waveform, an early peak and a late systolic peak were identified for derivation of early radial systolic blood pressure (rSBP) and late radial SBP (rSBP2), respectively. The central systolic blood pressure (cSBP) was then derived from rSBP2 as described previously [19]. A radial augmentation index (rAI) was calculated as [(rSBP2-DBP)/(rSBP-DBP)]× 100% and normalized to a heart rate of 75 beats/min.

### Submaximal exercise testing

Submaximal exercise testing was performed using a bicycle ergometer (Ergosana Schiller Semi-couch Safety Ergometer ERG 911 S/L, Swabian Alb, Germany). The subjects were asked to avoid caffeine-containing food or drinks and exercise on the day of study. Baseline assessment was performed after at least 5 min of rest. The initial workload was 25 W, followed by a stepwise increase workload of 25 W after each 2-min interval until heart rate reaches 70% or above the target heart rate, a maximum of 12 min, exhaustion, or achieving a maximum workload of 150 W.

### Statistical analysis

Results are presented as mean ± standard deviation. Demographic variables, echocardiographic indices, and arterial parameters of survivors and controls were compared by unpaired Student’s t test and Fisher’s exact test where appropriate. Analysis of variance with two factors was used to determine the effect of submaximal exercise stress (exercise vs at rest) or the effect of grouping (survivors vs controls) on the carotid arterial functional parameters. Pearson correlation analysis was used to assess associations between demographic and clinical variables and vascular functional parameters. Slopes of changes in vascular parameters with age between survivors and controls were compared by linear regression. Multiple linear regression of the entire cohort was performed to identify significant determinants of carotid IMT, baseline carotid stiffness index, and percentage increase in stiffness index during exercise. All statistical analyses were performed using SPSS version 22 (SPSS Inc., Chicago, IL, USA). A *p* value of < 0.05 was regarded as statistically significant.

## Results

### Subjects

Table 1 summarizes the demographic parameters of all subjects and clinical data of survivors. Ninety-six (54 males) survivors aged 25.0 ± 5.9 years were studied at 15.4 ± 5.9 years after completion of chemotherapy. The most common underlying diagnoses was acute lymphoblastic leukaemia, which occur in 50 (52%) of the 96 survivors. Of the 96 survivors, nine had relapse, five had cardiac irradiation, and one had undergone stem cell transplant. Details of dosages of chemotherapeutic drugs were not available in seven survivors. The mean cumulative anthracycline dose of the remaining 89 survivors was 258 ± 110 mg/m^2^ (range, 75 to 675 mg/m^2^). All of the survivors were free from cardiac symptoms and none were receiving cardiac medications at the time of study.

**Table 1:** Demographic, clinical, and vascular parameters

	Survivors (*n* = 96)	Controls (*n* = 60)	*p*
*Demographic parameters*			
Age (years)	25.0 ± 5.9	23.6 ± 5.8	0.16
Sex (M/F)	54/42	30/30	0.45
Weight (kg)	59.1 ± 10.8	59.2 ± 11.0	0.97
Height (cm)	166.1 ± 8.7	165.9 ± 8.6	0.85
Body mass index (kg/m^2^)	21.4 ± 3.2	21.4 ± 2.6	0.99
*Clinical parameters in survivors*			
Age at diagnosis (years)	8.0 ± 4.8		
Duration since completion of therapy (years)	15.4 ± 5.9		
Cumulative anthracycline dose (mg/m^2^)	258 ± 110 (range, 75–675)		
Diagnosis			
Acute lymphoblastic leukaemia	50		
Non-Hodgkin lymphoma	15		
Acute myeloid leukaemia	10		
Osteosarcoma	4		
Hodgkin lymphoma	5		
Wilm’s tumour	6		
Ewing Sarcoma	2		
Clear cell sarcoma of kidney	1		
Ganglioneuroblastoma	1		
Neuroblastoma	1		
Peripheral primitive neuroectodermal tumour	1		
Need for cardiac irradiation	5		
Relapse	9		
Cardiac medication	0		
*Vascular parameters*			
*Carotid arterial indices*			
IMT (mm)	0.44 ± 0.03	0.41 ± 0.02	< 0.001
Stiffness index	4.05 ± 0.02	3.85 ± 0.61	< 0.001
*Radial pulse waveform indices*			
Pulse pressure (mmHg)	42 ± 8	43 ± 9	0.34
cSBP (mmHg)	111 ± 16	105 ± 16	0.037
rAI (%)	64 ± 15	57 ± 13	0.004
rAI at 75 beats/min (%)	64 ± 14	58 ± 12	0.009

*cSBP* central systolic blood pressure, *IMT* intima-media thickness, *rAI* radial augmentation index

Sixty controls (30 males), aged 23.6 ± 5.8 years, were recruited and studied. The body weight, height, and body mass index were similar between survivors and controls (all *p* > 0.05).

### Baseline arterial parameters

The resting baseline arterial parameters of survivors and controls are shown in Table 1. Compared with controls, survivors had significantly greater carotid IMT (*p* < 0.001), carotid arterial stiffness index (*p* < 0.001), cSBP (*p* = 0.037), rAI (*p* = 0.004), and rAI adjusted for a heart rate of 75/min (*p* = 0.009).

**Fig. 1:**
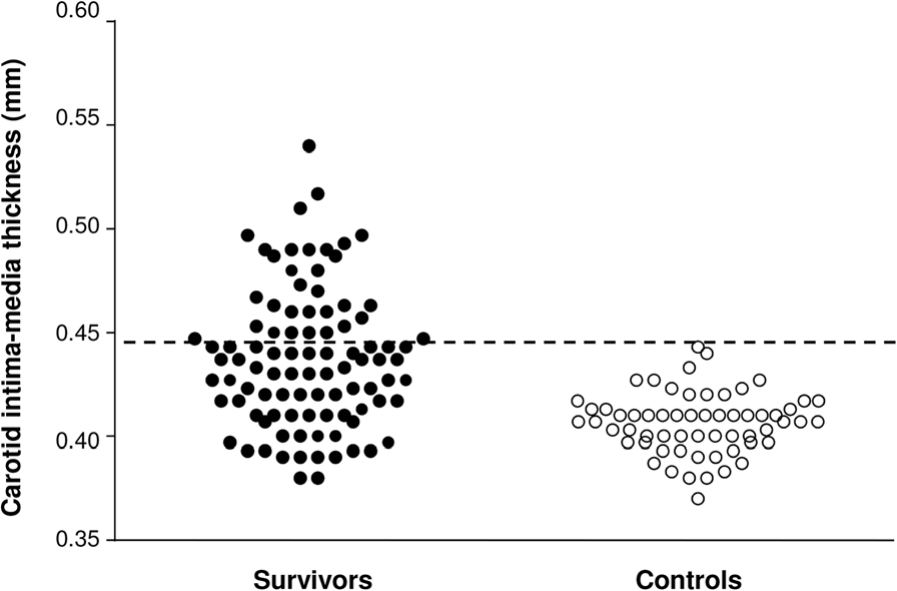
Scatter plots showing carotid intima-media thickness (IMT) in survivors and controls. The dash line represents the cutoff of 2 standard deviations above the mean IMT of controls

Using a carotid IMT cutoff of 0.499 mm, which represented 2 standard deviations above the mean of control subjects, 31 survivors (32%, confidence interval 23 to 43%) had increased carotid IMT (Fig. 1). Compared to survivors having normal carotid IMT, survivors with increased IMT were significantly older (27.8 ± 6.8 years vs 23.7 ± 5.0 years, p = 0.004), had a longer follow-up interval (17.5 ± 6.2 years vs 14.5 ± 5.6 years, *p* = 0.026), and had significantly greater rAI (70.68 ± 15.73% vs 60.97 ± 13.76%, *p* = 0.003) and rAI adjusted for a heart rate of 75/min (69.10 ± 16.11% vs 60.94 ± 12.34%, *p* = 0.007). There were, however, no differences between gender distribution, systemic blood pressures, and cumulative dose of anthracyclines between the two groups (all *p* > 0.05).

**Fig. 2:**
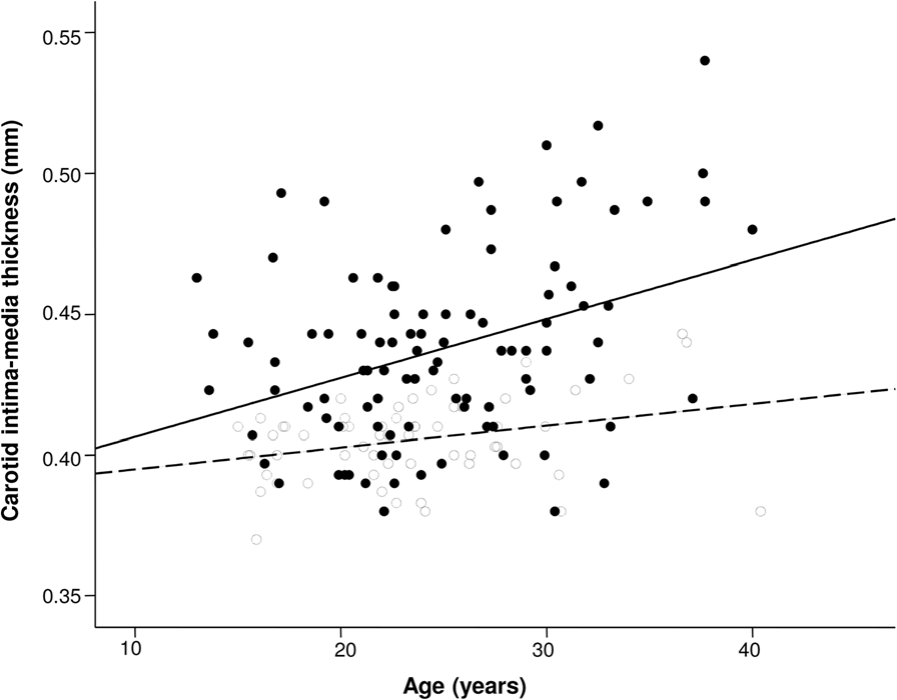
Scatter plot showing relationships between carotid intima-media thickness and age in survivors (solid circles) and controls (empty circles). The solid and dashed black lines represent the slope of change in carotid intima-media thickness with age in survivors and controls, respectively

Table 2 summarizes the univariate and multivariate analyses of the entire cohort for identification of significant correlates of carotid IMT. Univariate analysis showed that carotid IMT correlated positively with age (*r* = 0.35, *p* < 0.001), rAI (*r* = 0.41, *p* = 0 < 0.001), and adjusted rAI (*r* = 0.38, p < 0.001), but not with SBP, DBP, and cSBP (*p* > 0.05). Stratified by groups, the slope of increase in carotid IMT with age in survivors was significantly greater in survivors (2.10 ± 0.55 μm/year) than controls (0.77 ± 0.32 μm/year, *p* < 0.001) (Fig. 2).

**Table 2:** Univariate and multivariate analyses of carotid intima-media thickness

	Carotid IMT			
	r	p	β	p
Age at study	0.37	< 0.001	0.29	0.005
At rest				
SBP (mmHg)	−0.08	0.31	−0.03	0.77
DBP (mmHg)	0.11	0.18	0.08	0.42
cSBP (mmHg)	0.13	0.10	−0.04	0.66
rAI (%)	0.41	< 0.001	0.27	0.006
rAI at 75 beats/min (%)	0.38	< 0.001	−0.01	0.99
Carotid stiffness index	0.02	0.78	−0.07	0.44

All of the parameters were entered into the multivariate analysis. The correlation coefficient r for univariate analysis and β coefficient for multivariate analysis are presented

Abbreviations as in Table 1. *DBP* diastolic blood pressure, *SBP* systolic blood pressure

For stepwise multiple linear regression analyses, the independent covariates entered into the models included age, baseline systolic and diastolic blood pressures, cSBP, rAI, and rAI adjusted for a heart rate of 75/min. Significant independent correlates of carotid IMT were age (β = 0.27, *p* = 0.006) and rAI (β = 0.29, *p* = 0.005).

### Exercise arterial mechanics

Table 3 summarizes the carotid stiffness index and systemic blood pressures at rest and during submaximal exercise. At rest and during submaximal exercise, survivors had significantly greater carotid stiffness than controls (all *p* < 0.001), while systolic and diastolic blood pressures remained similar between the two groups (all *p* > 0.05).

**Table 3:** Carotid stiffness index and systemic blood pressure at rest and during submaximal exercise

	Rest		Exercise		p Value		
	Patients (*n* = 96)	Controls (*n* = 60)	Patients (*n* = 96)	Controls (*n* = 60)	*Group Factor*	*Exercise Factor*	*Interaction*
Carotid stiffness index	4.05 ± 0.67	3.85 ± 0.61	5.59 ± 1.31	4.13 ± 0.62	< 0.001	< 0.001	< 0.001
SBP (mmHg)	119 ± 12	119 ± 12	155 ± 17	153 ± 16	0.52	< 0.001	0.77
DBP (mmHg)	72 ± 8	71 ± 8	80 ± 12	77 ± 9	0.09	< 0.001	0.40

Abbreviations as in Table 1 and Table 2

**Fig. 3:**
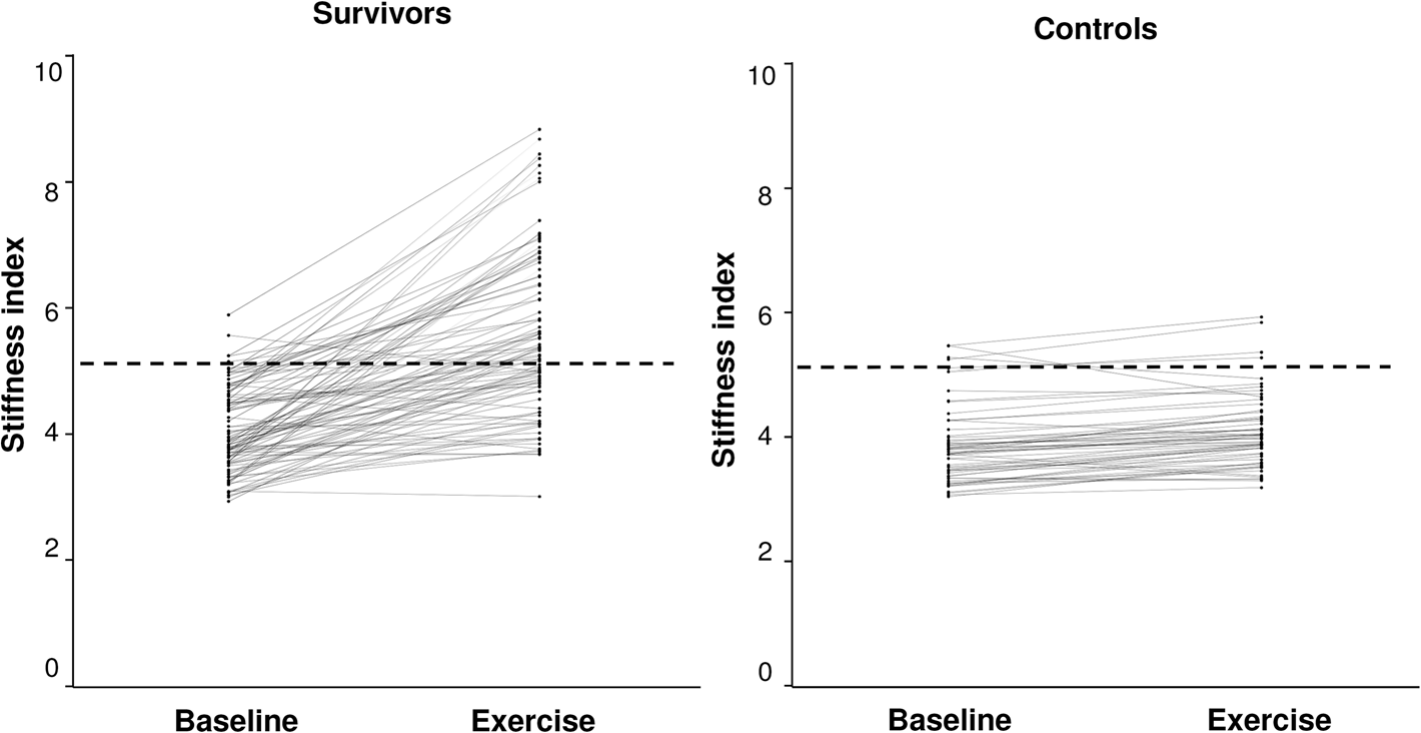
Stiffness index at baseline and during submaximal exercise in survivors and controls. The dashed lines represent the cutoff of 2 standard deviations above the mean stiffness index at submaximal exercise of controls

**Fig. 4:**
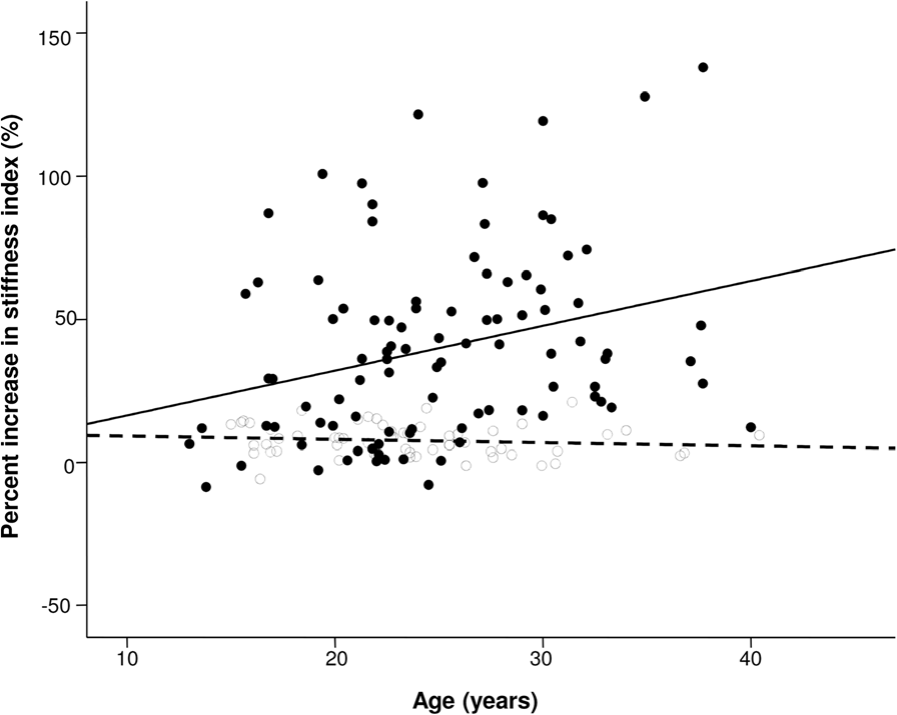
Scatter plot showing relationships between percent increase in stiffness index and age in survivors (solid circles) and controls (empty circles). The solid and dashed black lines represent the slope of percentage change in stiffness index with age in survivors and controls, respectively

Carotid arterial stiffness index increased significantly during exercise in both groups (*p* < 0.001 for exercise factor) (Fig. 3). Significant interactions between group and exercise factors for carotid stiffness index suggested differences in magnitude of changes with exercise between the two groups (Table 3), which suggested a significantly greater change in arterial stiffness during exercise in survivors compared with controls. The percentage change in carotid stiffness index from baseline to submaximal exercise was significantly higher in survivors than controls (39.9 ± 32.5% vs 7.5 ± 5.5%, *p* < 0.001). Stratified by groups, the slope of change in percentage increase in stiffness index with age in survivors was significantly greater in survivors (1.59 ± 0.55%/year) than controls (− 0.12 ± 0.12%/year, *p* < 0.001) (Fig. 4). On the other hand, the percentage changes in systolic (30.4 ± 12.4% vs 29.8 ± 13.0%, *p* = 0.80) and diastolic (11.5 ± 14.3% vs 8.6 ± 9.8%, *p* = 0.14) blood pressures were similar between the two groups.

Using an exercise stiffness index cutoff of 5.35, which represented 2 standard deviations above the mean of control subjects during submaximal exercise, 51 survivors (53%, 95% confidence intervals 43 to 63%) had increased carotid stiffness during exercise. Survivors with increased exercise stiffness index compared to those without had significantly greater resting stiffness index (4.26 ± 0.71 vs 3.83 ± 0.55, *p* = 0.001). There were, however, no differences in age, gender distribution, cumulative dose of anthracyclines carotid IMT, systemic blood pressures at rest and during exercise, and radial arterial waveform parameters between the two subgroups (all *p* > 0.05).

### Correlates of baseline and exercise carotid parameters

Table 4 summarizes the results of univariate and multivariate correlation analyses of the entire cohort with regard to baseline and dynamic changes in carotid stiffness index. Baseline stiffness index correlated with gender (p = 0.001), systolic blood pressure at rest (*p* < 0.001) and at submaximal exercise (*p* = 0.002). Stepwise multiple linear regression analysis identified age (β = 0.22, *p* = 0.003), gender (β = − 0.18 being female, *p* = 0.021), and resting systolic (β = 0.59, *p* < 0.001) and diastolic (β = − 0.53, *p* < 0.001) blood pressures as independent significant determinants of baseline stiffness index.

**Table 4:** Univariate and multivariate analyses of arterial stiffness index and exercise-induced changes

	Baseline stiffness index				Percentage increased in stiffness index during exercise			
	r	p	β	p	r	p	β	p
Age at study	0.08	0.31	0.22	0.003	0.24	0.002	0.15	0.07
Gender	−0.27	0.001	− 0.18	0.021	0.02	0.80	−0.01	0.95
At rest								
SBP	0.29	< 0.001	0.59	< 0.001	−0.08	0.31	−0.06	0.47
DBP	−0.07	0.36	−0.53	< 0.001	0.12	0.14	0.09	0.27
cSBP	0.02	0.81	−0.09	0.29	0.07	0.36	0.03	0.67
Adjusted rAI	−0.15	0.06	0.07	0.42	0.21	0.009	0.10	0.22
Carotid IMT	0.02	0.78	0.08	0.29	0.32	< 0.001	0.32	< 0.001
At submaximal exercise								
SBP	0.25	0.002	0.13	0.14	0.01	0.89	0.02	0.77
DBP	−0.01	0.93	−0.03	0.71	−0.03	0.74	−0.08	0.29

Abbreviations as in Table 1 and Table 2

All of the parameters were entered into the multivariate analysis. The correlation coefficient r for univariate analysis and β coefficient for multivariate analysis are presented

The dynamic percentage increase in stiffness index during exercise was correlated positively with age (*p* = 0.002), adjusted rAI (*p* = 0.009), and carotid IMT (*p* < 0.001) (Fig. 4) in univariate analysis. On the other hand, stepwise multiple linear regression analysis revealed carotid IMT (β = 0.32, *p* < 0.001) as the only significant independent determinant.

In cancer survivors, there were no correlations between baseline stiffness index and age at diagnosis, years after completion of anthracycline therapy, and the cumulative anthracycline dose received (all *p* > 0.05). The exercise stiffness index correlated with age (*r* = 0.27, *p* = 0.008) and years after completion of anthracycline therapy (*r* = 0.26, *p* = 0.013), but not the age at diagnosis or the cumulative anthracycline dose received (both *p* > 0.05). Similarly, the dynamic percentage increase in stiffness index correlated with age at study (*r* = 0.29, *p* = 0.005), and tended to correlated with years after completion of anthracycline therapy (*r* = 0.21, *p* = 0.053).

## Discussion

The present study shows an increase in carotid arterial stiffness, central SBP, radial augmentation index, and carotid IMT in anthracycline-treated long-term survivors of childhood cancers. Furthermore, worsening of arterial stiffness was evident during submaximal exercise, with about half of the survivors having an arterial stiffness index exceeding the upper limit of normal. Age amongst other factors is independently associated with baseline and exercise stiffness index in survivors. The important novel finding of this study is the steeper slope of increase in both carotid IMT and exercise-induced changes in carotid arterial stiffness with age in survivors compared with that in controls, implicating the possibility of accelerated vascular ageing in adult survivors of childhood cancers.

Studies on arterial mechanics in cancer survivors are limited to non-stressed, short- to intermediate-term assessment after completion of chemotherapy. Chaosuwannakit et al. reported an increase in aortic stiffness in adults receiving anthracycline for the treatment of breast cancer, lymphoma, or leukaemia within 4 months of chemotherapy exposure [11]. Mizia-Stec et al. similarly found decreased compliance and increased stiffness of the aortic and carotid arteries in breast cancer patients assessed at 6 months after the last dose of anthracycline [20]. However, longer term studies in adults are not available. In children and adolescent cancer survivors, previous studies have shown increased aortic stiffness, as assessed by echocardiography [21] and measurement of aortic pulse wave velocity [22, 23], and increased carotid arterial stiffness [24]. Whether the change in arterial mechanics in childhood cancer survivors would persist in adulthood has been unclear. Our data provide additional evidence to suggest that adult survivors of childhood cancers indeed continue to have altered arterial mechanics as characterized by increased carotid arterial stiffness, increased radial augmentation index, and increased central SBP.

In this study, we have further defined the exercise arterial mechanics in long-term survivors. Assessment of arterial mechanics during exercise stress has recently gained increasing interest. Moon et al. recently showed the unmasking of increase in arterial stiffness by isometric handgrip exercise in patients with coronary artery disease [12]. High-intensity acute exercise has further been shown to increase carotid-femoral pulse wave velocity, a robust marker of central arterial stiffness, in patients with untreated recently diagnosed grade I essential hypertension but not in healthy subjects [25]. Our group has further reported on potential deleterious effect of augmented arterial stiffness during exercise on dilation of the neoaorta after arterial switch operation [13]. The findings in the present study of an augmented increase in carotid arterial stiffness with levels exceeding normal in about half of the survivors may hence have important haemodynamic and clinical implications as discussed later.

Apart from functional alteration of the arteries at rest and during exercise, structural changes with an increase in carotid IMT, the surrogate marker of atherosclerosis, was found in about one-third of the survivors. Most of the previous adult [26, 27] and paediatric [28] studies have primarily documented an increase in carotid IMT in patients with head and neck irradiation. An acute increase in carotid IMT has also been reported to occur within 10 weeks after completion of cisplatin-based chemotherapy in patients with testicular cancer [29]. Our findings are of clinical significance given the extended duration of follow-up and that 95% (89/94) of our survivors had no history of irradiation. Additionally, the novel finding of a steeper slope of increase in carotid IMT with age in survivors compared with that in healthy controls implies accelerated vascular ageing with perhaps an increased risk of premature atherosclerosis. While this undoubtedly remains speculative, the excess cardiac mortality [6–8] and excess risk of cerebrovascular events survivors [9] with cancers diagnosed during childhood and young adulthood may be clues to an underlying vasculopathy related to cancer treatments.

While the exact mechanisms that underlie the functional and structural alterations of the arteries in adult survivors of childhood cancers remains to be confirmed, several lines of evidence suggest that endothelial dysfunction might be the culprit. In vitro studies have demonstrated that doxorubicin [30, 31] induces apoptosis of endothelial cells in vitro, which may impair endothelial-dependent relaxation [31]. The generation of oxygen free radicals [32, 33] and induction of inflammatory cytokines [34, 35] by anthracyclines may mediate the endothelial damage. In paediatric cancer patients, Chow et al. have shown significant reduction of endothelium-dependent brachial arterial dilation during reactive hyperaemia [36]. The role of endothelial dysfunction in atherosclerosis [37] and increasing the vasomotor tone, and hence arterial stiffness [38], are well documented. The augmented increase in arterial stiffness with exercise in survivors further reflects structural changes within the vascular matrix, which may be caused by nuclear actions of anthracyclines [39], oxidative stress [33], and senescence of the vascular smooth muscle cells [40]. Data on the relationship between arterial stiffness and cumulative dose of anthracycline are, however, conflicting [41]. In the present study, we did not find significant correlations between the anthracycline dose and the structural and function vascular parameters, implicating that anthracycline-induced vascular changes may not be a dose-dependent phenomenon.

Our findings have several clinical implications. Firstly, stiffening of the arteries, as reflected by the cross-sectional carotid stiffness index and radial augmentation index, and its augmentation during exercise stress increase ventricular afterload and potentiate the development of ventricular dysfunction. Using speckle tracking echocardiography, we have previously shown subclinical impairment of systolic [2] and diastolic [42] ventricular deformation even in survivors with preserved left ventricular ejection fraction. Roche et al. further found blunting of left ventricular force-frequency relationship indicating impairment of systolic contractile reserve during exercise in childhood cancer survivors [43]. Indeed, abnormal ventricular-arterial coupling has recently been shown to be strongly predictive of cancer therapeutics-related cardiac dysfunction in breast cancer patients, and that this abnormality appeared to be driven primarily by an increased arterial elastance [44]. Secondly, arterial stiffening is possibly a harbinger of increased risk of hypertension. Data from the Baltimore Longitudinal Study of Aging suggest that arterial stiffness is independent predictors of longitudinal increase in systolic hypertension in normotensive individuals [45]. Our findings of systolic blood pressure being an independent correlate of arterial stiffness at rest and during submaximal exercise, and that radial arterial waveform-derived central SBP is increased in our survivors lend support to this proposition. Of concern is that the occurrence of hypertension in childhood cancer survivors has been shown to significantly increase the risk of major cardiac events [46]. Thirdly, amplification of vascular dysfunction with age is reflected the steeper slope of increase in carotid IMT with age in survivors and that age being an independent correlate of the absolute and the percentage increase in exercise stiffness index. The inherent risks associated with vascular dysfunction are expected to increase similarly with ageing. Increased vigilance in monitoring for development premature cardiovascular and cerebrovascular diseases is hence warranted.

Several limitations to this study warrant comments. Firstly, this is a cross-sectional study that evaluated a single time point long-term after completion of chemotherapy during childhood. It would have been ideal to track the changes of vascular parameters and to determine the longitudinal trajectory in each of the subjects. Secondly, the superimposed influence of irradiation therapy on vascular mechanics was not ascertained in this study as only 5% of our survivors had irradiation therapy. Thirdly, we did not formally assess ventricular-arterial interaction in our subjects. As alluded to earlier, abnormal ventricular-arterial coupling may help to predict the development of cardiac dysfunction in survivors of cancers [44]. Further studies to assess the coupling during exercise stress may perhaps even be more revealing.

In conclusion, arterial dysfunction is evident in adult survivors of childhood cancer and appears to worsen with age. Their carotid arterial stiffness is increased at rest and worsened during submaximal exercise About one-third of the long-term survivors have structural alteration of the arteries with increased carotid IMT and about half have an arterial stiffness index exceeding the upper limit of normal during submaximal exercise. Further studies to explore the consequences of premature vascular ageing and adverse ventricular-vascular interaction in this at risk population are warranted.
